# Correction: Romero et al. Effect of Hypoxia in the Transcriptomic Profile of Lung Fibroblasts from Idiopathic Pulmonary Fibrosis. *Cells* 2022, *11*, 3014

**DOI:** 10.3390/cells15131193

**Published:** 2026-06-30

**Authors:** Yair Romero, Yalbi Itzel Balderas-Martínez, Miguel Angel Vargas-Morales, Manuel Castillejos-López, Joel Armando Vázquez-Pérez, Jazmín Calyeca, Luz María Torres-Espíndola, Nelly Patiño, Angel Camarena, Ángeles Carlos-Reyes, Edgar Flores-Soto, Guadalupe León-Reyes, Martha Patricia Sierra-Vargas, Iliana Herrera, Erika Rubí Luis-García, Víctor Ruiz, Rafael Velázquez-Cruz, Arnoldo Aquino-Gálvez

**Affiliations:** 1 Facultad de Ciencias, Universidad Nacional Autónoma de México (UNAM), Mexico City 04510, Mexico; yair@ciencias.unam.mx; 2 Laboratorio de Biología Computacional, Instituto Nacional de Enfermedades Respiratorias Ismael Cosío Villegas (INER), Mexico City 14080, Mexico; yalbibalderas@gmail.com; 3 Laboratorio de Biología Molecular, Departamento de Fibrosis Pulmonar, Instituto Nacional de Enfermedades Respiratorias Ismael Cosío Villegas (INER), Mexico City 14080, Mexico; vargas.qx03@gmail.com (M.A.V.-M.); vicoruz@yahoo.com.mx (V.R.); 4 Departamento de Epidemiología y Estadística, Instituto Nacional de Enfermedades Respiratorias Ismael Cosío Villegas (INER), Mexico City 14080, Mexico; mcastillejos@gmail.com; 5 Laboratorio de Biología Molecular de Enfermedades Emergentes y EPOC, Instituto Nacional de Enfermedades Respiratorias Ismael Cosío Villegas (INER), Mexico City 14080, Mexico; joevazpe@gmail.com; 6 Division of Pulmonary, Critical Care and Sleep Medicine, Department of Internal Medicine, Davis Heart and Lung Research Institute, The Ohio State University, Columbus, OH 43210, USA; calyecajaz@gmail.com; 7 Laboratorio de Farmacología, Instituto Nacional de Pediatría (INP), Mexico City 04530, Mexico; luzmtorres@gmail.com; 8 Unidad de Citometría de Flujo (UCiF), Instituto Nacional de Medicina Genómica (INMEGEN), Mexico City 14610, Mexico; lnpatino@inmegen.gob.mx; 9 Laboratorio de HLA, Instituto Nacional de Enfermedades Respiratorias Ismael Cosío Villegas (INER), Mexico City 14080, Mexico; ang_edco@yahoo.com.mx; 10 Laboratorio de Onco-Inmunobiología, Departamento de Enfermedades Crónico-Degenerativas, Instituto Nacional de Enfermedades Respiratorias Ismael Cosío Villegas (INER), Mexico City 14080, Mexico; reyes_cardoso@yahoo.com; 11 Departamento de Farmacología, Facultad de Medicina, Universidad Nacional Autónoma de México (UNAM), Mexico City 04510, Mexico; edgarfloressoto@yahoo.com.mx; 12 Laboratorio de Genómica del Metabolismo Óseo, Instituto Nacional de Medicina Genómica (INMEGEN), Mexico City 14610, Mexico; greyes@inmegen.gob.mx; 13 Departamento de Investigación en Toxicología y Medicina Ambiental, Instituto Nacional de Enfermedades Respiratorias Ismael Cosío Villegas (INER), Mexico City 14080, Mexico; pat_sierra@yahoo.com; 14 Laboratorio de Biología Celular, Departamento de Fibrosis Pulmonar, Instituto Nacional de Enfermedades Respiratorias Ismael Cosío Villegas (INER), Mexico City 14080, Mexico; iliany23@yahoo.com.mx (I.H.); erikarubi.84@gmail.com (E.R.L.-G.)

## Text Correction

An error occurred in the original publication [1] due to incorrect identification of the hypoxia and normoxia samples, resulting from an erroneous assignment of experimental conditions in our bioinformatics process. Since all subsequent steps—differential expression analysis, expression change estimation (log_2_), and metabolic pathway enrichment—depended on this metadata, the underlying read counts and statistical significance for each gene are correct, but the two conditions were systematically interchanged. Consequently, it was necessary to swap the terms “upregulated” and “downregulated” in the affected passages of the corrected version. We sincerely regret this error and offer our deepest apologies for any confusion or inconvenience it may have caused our readers and the scientific community. We remain firmly committed to maintaining the accuracy of the scientific record. To ensure clarity, the specific corrections are detailed below.

### Abstract

Abstract: Idiopathic pulmonary fibrosis (IPF) is an aging-associated disease characterized by exacerbated extracellular matrix deposition that disrupts oxygen exchange. Hypoxia and its transcription factors (HIF-1α and 2α) influence numerous circuits that could perpetuate fibrosis by increasing myofibroblast differentiation and by promoting extracellular matrix accumulation. Therefore, this work aimed to elucidate the signature of hypoxia in the transcriptomic circuitry of IPF-derived fibroblasts. To determine this transcriptomic signature, a gene expression analysis with six lines of lung fibroblasts under normoxia or hypoxia was performed; three cell lines were derived from patients with IPF, and three were from healthy donors, obtaining a total of 36 replicates. We used the Clariom D platform, which allows us to evaluate a huge number of transcripts, to analyze the response to hypoxia in both controls and IPF. The controls’ response is greater by the number of genes and complexity. In the search for specific genes responsible for the IPF fibroblast phenotype, nineteen dysregulated genes were found in lung fibroblasts from IPF patients in hypoxia (nine down-regulated and ten up-regulated). In this sense, the signaling pathways revealed to be affected in the pulmonary fibroblasts of patients with IPF may represent an adaptation to chronic hypoxia.

### Results, 3.1. Transcriptional Response to Hypoxia in Control and IPF-Derived Lung Fibroblasts, Paragraph 4

Network analysis using Ingenuity Pathway Software (IPA) shows a transcriptomic landscape in normal fibroblasts during hypoxia revealing metabolic changes regulated by HIF-1α (Figure 3A,B). These changes showed a central role of HIF-1α in YTH N6-Methyladenosine RNA Binding Protein 2 (YTHDF2)-mediated cell cycle regulation which promotes mRNA decay during the cell cycle in somatic reprogramming (Figure 3A). Consistent with that, TP53, a master cell cycle regulator implicated in aging, senescence, and fibrosis, was down-regulated (Figure 3C). The radial diagram of TP53 and its targets reveals its central role in the regulation of autophagy-related processes (ATG4a and ATG4b), topological stress (TOP2B), glycolysis (TIGAR), and insulin-like growth factor regulation (IGF) and transport and uptake by insulin-like growth factor binding proteins (PAPPA). In summary, controls have a complex network involving more genes in the hypoxia adaptation and antagonistic mechanisms such as autophagy regulation. IPA also identified alterations in the AKT signaling in IPF; AKT can negatively regulate autophagy in the opposite direction to controls (Figure 3D).

### Results, 3.3. The Transcription Signature of Hypoxia in IPF Fibroblasts, Paragraph 1

A third Venn diagram was constructed to identify the transcriptional signature with dysregulated genes shared between the controls and IPF (Figure 7A). Of the three groups of genes analyzed, the first group contained genes that belong to the adaptation hypoxia response in controls (311); the analysis coincides with those that were made as a whole by condition and cell line. As these pathways are not found in IPF fibroblasts, they provide us with information that could help elucidate the mechanisms of IPF pathogenesis since, for some reason, they have been lost or blocked. In the second group, only 35 genes were found altered in both; these genes are involved in hypoxia and mesenchymal pathways. Finally, the genes that are induced by hypoxia are shared in different cell lines from IPF. Tables 2 and 3 show the 19 DEGs, divided into up- and down-regulated; there are some similarities in the four up-regulated genes in our study because previous studies in IPF report them up-regulated, and this is the case with Coagulation factor III (F3), Hedgehog interacting protein (HHIP), Interleukin 6 (IL-6), and Stanniocalcin 1 (STC1) (Table 2).

### Results, 3.3. The Transcription Signature of Hypoxia in IPF Fibroblasts, Paragraph 2

Five genes were identified to be up-regulated, that have not been studied in IPF, which are Cyclin G2 (CCNG2), Potassium Channel Tetramerization Domain Containing 16 (KCTD16), Basic helix-loop-helix family member e41 (BHLHE41), Syntaxin binding protein 6 (STXBP6), and Serpin family B member 7 (SERPINB7) (Table 2).

### Results, 3.3. The Transcription Signature of Hypoxia in IPF Fibroblasts, Paragraph 3

Nine genes were found down-regulated, of which four genes have been previously related to IPF: Endothelial PAS domain protein 1 (EPAS1), Transferrin receptor (TFRC), Endothelin receptor type A (EDNRA) and Periostin (POSTN). The other five genes have not been associated with IPF: the Alcohol dehydrogenase 1B (ADH1B), Protocadherin 18 (PCDH18), Homeobox A5 (HOXA5), Solute carrier family 14-member 1 (SLC14A1), and Ubiquitin specific peptidase 18 (USP18) (Table 3).

### Conclusions

Pathways in IPF fibroblasts that are not shared with healthy cells are pathways that could help us begin to guide potential therapeutic targets. In the case of fibroblasts from patients with IPF, low levels of EPAS (HIF-2α) suggest that it plays an essential role in this chronic adaptation to hypoxia.

## Error in Figure

In the original publication, there was a mistake in Figure 2A–D, as published. As up or down regulation is inverted. The corrected Figure 2 appears below. We revised the Figure 2 legend in response to the Academic Editor’s comments. As part of this revision and following the Academic Editor’s suggestion, we added the new Supplementary Figures S4 and S5 (heatmaps of differentially expressed genes with gene symbols), which were not included in the original publication.

### Correction in Legend of Figure 3

In the original publication, the legend of Figure 3 incorrectly described the direction of gene regulation because the hypoxia and normoxia samples had been interchanged during data processing; consequently, the up-regulated and down-regulated genes were inverted. In the corrected version, the regulation directions—and the corresponding node colors (green = up-regulated, red = down-regulated)—have been reassigned to match the proper hypoxia-versus-normoxia comparison, while the network topology and all other elements remain unchanged. The correct legend appears below.

**Figure 3.** Networks in response to hypoxia in control and IPF fibroblasts. Radial diagrams of HIF1 surrounded by its targets, (**A**) control fibroblasts and (**B**) IPF fibroblasts. (**C**) Radial diagram of TP53 surrounded by its targets in control fibroblasts. (**D**) Radial diagram of AKT surrounded by its targets in IPF fibroblasts. Colored nodes refer to genes in our dataset (green up-regulated; red down-regulated). Uncolored nodes were not identified as differentially expressed in our experiment and were integrated into the computationally generated IPA networks. Arrows identify predicted relationships (orange leads to inhibition, blue leads to activation, yellow finds inconsistency with downstream molecules, and gray has no effect predicted).

### Correction in Legend of Figure 4

In the original publication, the legend of Figure 4 incorrectly described the direction of gene regulation because the hypoxia and normoxia samples had been interchanged during data processing; consequently, the up-regulated and down-regulated genes were inverted. In the corrected version, the regulation directions—and the corresponding node colors (green = up-regulated, red = down-regulated)—have been reassigned to match the proper hypoxia-versus-normoxia comparison, while the network topology and all other elements remain unchanged. The correct legend appears below.

**Figure 4.** HIF-1α Signaling Pathways in control Fibroblasts. Colored nodes refer to differentially expressed genes found in our dataset control fibroblasts hypoxia vs. normoxia (green up-regulated; red down-regulated). Uncolored nodes were not identified as differentially expressed in our experiment and were integrated into the computationally generated IPA networks. This figure was created with Ingenuity Pathway Analysis, Version 2000–2022 QIAGEN. For further information about the symbols, please go through their web page: https://qiagen.my.salesforce-sites.com/KnowledgeBase/articles/Knowledge/Legend.

### Correction in Legend of Figure 5

In the original publication, the legend of Figure 5 incorrectly described the direction of gene regulation because the hypoxia and normoxia samples had been interchanged during data processing; consequently, the up-regulated and down-regulated genes were inverted. In the corrected version, the regulation directions—and the corresponding node colors (green = up-regulated, red = down-regulated)—have been reassigned to match the proper hypoxia-versus-normoxia comparison, while the network topology and all other elements remain unchanged. The correct legend appears below.

**Figure 5.** HIF1α signaling pathways in fibroblasts from idiopathic pulmonary fibrosis. Colored nodes refer to differentially expressed genes found in our dataset of IPF fibroblasts under hypoxia vs. normoxia (green up-regulated; red down-regulated). Uncolored nodes were not identified as differentially expressed in our experiment and were integrated into the computationally generated IPA networks. This figure was created with Ingenuity Pathway Analysis, Version 2000–2022 QIAGEN. For further information about the symbols, please go through their web page: https://qiagen.my.salesforce-sites.com/KnowledgeBase/articles/Knowledge/Legend.

### Error and Addition in Supplementary Figures

In the original publication, labels were inverted. The corrected Supplementary Figures S2 and S3 and new Supplementary Figures S4 and S5 attached. Supplementary Materials Section should be: The following supporting information can be downloaded at: https://www.mdpi.com/article/10.3390/cells11193014/s1, Figure S1: Contrasts matrix. Figure S2: Principal Component Analysis by condition. Figure S3: Principal Component Analysis by cell lines. Figure S4. Heatmaps of differentially expressed genes (DEGs). In all panels, rows represent DEGs (adj. *p*-value < 0.05, |log_2_FC| ≥ 1, NM_ accessions only) clustered by Euclidean distance (Ward’s D2 method). Color intensity reflects row-normalized z-scores (blue = down-regulated; red = up-regulated). Top annotation bars indicate cell line identity and experimental condition (orange = hypoxia; purple = normoxia). (**A**) Control fibroblasts (1007 DEGs; C1–C3), same data as Figure 2C, with gene symbols on the y-axis. (**B**) IPF fibroblasts (242 DEGs; F1–F3), same data as Figure 2D, with gene symbols on the y-axis. (**C**) Same as A, with columns ordered by replicate number (H1 from all cell lines, then H2, H3, N1, N2, N3) without column clustering, to address the Editor’s suggestion. (**D**) Same as B, with columns ordered by replicate number without column clustering. The consistent separation between hypoxia and normoxia demonstrates the robustness of the transcriptomic response regardless of sample arrangement. Figure S5. Heatmaps of differentially expressed genes (DEGs) with gene symbols on the y-axis. In both panels, rows represent DEGs (adj. *p*-value < 0.05, |log_2_FC| ≥ 1, NM_ accessions only) clustered by Euclidean distance (Ward’s D2 method). Color intensity reflects row-normalized z-scores (blue = down-regulated; red = up-regulated). Top annotation bars indicate cell line identity and experimental condition (orange = hypoxia; purple = normoxia). (**A**) Control fibroblasts (1007 DEGs; C1–C3), same data as Figure 2C. Due to the large number of genes, font size has been reduced to accommodate all labels. (**B**) IPF fibroblasts (242 DEGs; F1–F3), same data as Figure 2D. Complete gene lists with expression statistics are provided in Supplementary Table S1. Tables S1: Gene Ontology (GO) of the study groups. 

## Correction in Tables

### Error in Table 2

In the original publication, the title of Table 2 and the normoxia (N) and hypoxia (H) expression columns were inverted because the hypoxia and normoxia samples had been interchanged during data processing. The classification of the genes as up-regulated and the values reported in the N and H columns have been corrected accordingly, whereas the gene list, descriptions, models, and references remain unchanged. The correct title and table content appears below:

**Table 2:** List of up-regulated genes in lung fibroblasts from IPF patients in normoxia and hypoxia.

Gene	Also Known as	N	H	OS	Description	Models	References
F3	TF; AGT; CD142	NS	Up	Up	Coagulation factor III is a surface receptor, and it participates in the coagulation cascade	IPF-HLF	[27]
HHIP	HIP	NS	Up	Up	Hedgehog interacting protein is involved with Hedgehog signaling pathway in embryonic development	IPF-HLF	[28,29]
IL6	CDF; HGF; HSF; BSF2; IL-6; BSF-2; IFNB2; IFN-β-2	NS	Up	Up	Interleukin 6 encodes for a cytokine with inflammatory functions	IPF-HLF and BMM	[30,31]
STC1	STC	NS	Up	Up	Stanniocalcin 1 encodes for homodimeric glycoprotein with paracrine and autocrine functions	Increased in plasma of patients with IPF	[32]
DDIT4	Dig2; REDD1; REDD-1	NS	Up	-	DNA Damage Inducible Transcript 4. Gene related to response to virus, hypoxia, DNA damage, and tumor regulation	Expression associated with lncRNAs in IPF	[33]
CCNG2	-	NS	Up	-	Encodes for cyclin-G2 involved in cell cycle	NS-IPF	-
KCTD16	-	NS	Up	-	Potassium channel tetramerization domain containing 16 regulates GABA receptor signaling	NS-IPF	-
BHLHE41	DEC2; FNSS1; hDEC2; BHLHB3; SHARP1	NS	Up	-	Basic helix-loop-helix family member e41, involved in circadian rhythm and cell differentiation	NS-IPF	-
STXBP6	amisyn; HSPC156	Up	Up	-	Syntaxin binding protein 6 is involved in regulating SNARE complex formation	NS-IPF	-
SERPINB7	PPKN; TP55; MEGSIN	NS	Up	-	Serpin family B member 7 encodes for a protein that functions as a protease inhibitor	NS-IPF	-

N = Normoxia results in this study; H = Hypoxia results in this study; OS = Other Studies; IPF-HLF = IPF human lung fibroblast; BMM = Bleomycin mouse model; BAL = Bronchoalveolar lavage; NS-IPF = Not studied in idiopathic pulmonary fibrosis; Non-significant = NS. The search for the function of the genes was carried out using the gene card (https://www.genecards.org/). As for the studies reported in IPF, a basic search was performed in PubMed with the name of the gene or protein it encodes (https://pubmed.ncbi.nlm.nih.gov/), and only those that seemed relevant were considered.

### Error in Table 3

In the original publication, the title of Table 3 and the normoxia (N) and hypoxia (H) expression columns were inverted because the hypoxia and normoxia samples had been interchanged during data processing. The classification of the genes as down-regulated and the values reported in the N and H columns have been corrected accordingly, whereas the gene list, descriptions, models, and references remain unchanged. The correct title and table content appears below:

**Table 3:** List of down-regulated genes in lung fibroblasts from IPF patients in normoxia and hypoxia.

Gene	Also Known as	N	H	OS	Description	Models	References
EPAS1	HLF; MOP2; ECYT4; HIF2A; PASD2; bHLHe73	Down	Down	Up	Endothelial PAS domain protein 1 is a gene that encodes a transcription factor involved in signaling pathway in hypoxia	IPF-HLF	[9]
TFRC	CD71, IMD46, T9, TFR, TFR1, TR, TRFR, p90	Down	Down	Up	Transferrin receptor encodes a cell surface receptor associated with cellular iron uptake and is required for erythropoiesis	IPF-HLF, BMM and BAL of IPF patients	[34,35]
POSTN	PN; OSF2; OSF-2; PDLPOSTN	Down	Down	Up	Gen encodes for periostin protein with functions in tissue development and regeneration. Expression related to IPF progression	IPF-HLF and BMM	[36,37]
EDNRA	ETA; ET-A; ETAR; ETRA; MFDA; ETA-R; hET-AR	NS	Down	Up	Endothelin receptor type A encodes an endothelin-1 receptor with vasoconstriction properties	Primary rat alveolar type II cells	[38,39]
HOXA5	HOX1; HOX1C; HOX1.3	NS	Down	Up	Homeobox A5 encodes for transcription factors called homeobox genes spatially and temporally regulated during embryonic development	NS-IPF	-
PCDH18	PCDH68L	NS	Down	Down	Protocadherin 18 encodes for a protein member of the subfamily of cadherin superfamily related to cell–cell connections	NS-IPF	-
ADH1B	ADH2; HEL-S-117	NS	Down	-	Alcohol dehydrogenase 1B encodes for a protein member of the alcohol dehydrogenase family	NS-IPF	-
SLC14A1	JK; UT1; UTE; HUT11; Jk(a); Jk(b); RACH1; RACH2; UT-B1; HUT11A; HsT1341	NS	Down	-	Solute carrier family 14-member 1 encodes for a protein membrane transporter that mediates urea transport in erythrocytes	NS-IPF	-
USP18	ISG43; UBP43; PTORCH2	NS	Down	-	Ubiquitin specific peptidase 18 encodes for an enzyme that belongs to ubiquitin-specific proteases family	NS-IPF	

N = Normoxia results in this study; H = Hypoxia results in this study; OS = Other Studies; IPF-HLF = IPF human lung fibroblast; BMM = Bleomycin mouse model; BAL = Bronchoalveolar lavage; NS-IPF = Not studied in idiopathic pulmonary fibrosis; Non-significant = NS. The search for the function of the genes was carried out using the gene card (https://www.genecards.org/). As for the studies reported in IPF, a basic search was performed in PubMed with the name of the gene or protein it encodes (https://pubmed.ncbi.nlm.nih.gov/), and only those that appeared relevant were considered.

### Error in Supplementary Table S1

In the original publication, labels are inverted. The corrected supplementary table is attached as Supplementary_TableS1_v2.0.xlsx.

The authors state that the main scientific conclusions are unaffected. This correction was approved by the Academic Editor. The original publication has also been updated.

## Figures and Tables

**Figure 2:**
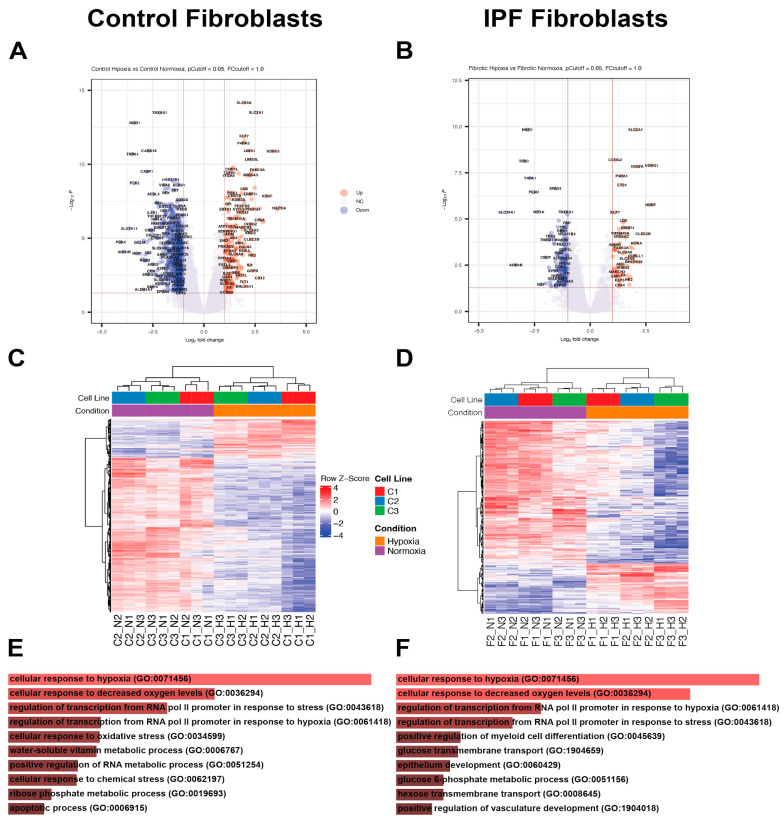
Transcriptomic response of lung fibroblasts to hypoxia. (**A**) Volcano plot of differentially expressed genes (DEGs) in control fibroblasts (hypoxia vs. normoxia). (**B**) Volcano plot of DEGs in IPF fibroblasts (hypoxia vs. normoxia). (**C**) Heatmap of 1007 DEGs in control fibroblasts (C1–C3) under hypoxia (H) and normoxia (N) conditions. (**D**) Heatmap of 242 DEGs in IPF fibroblasts (F1–F3) under hypoxia (H) and normoxia (N) conditions. In panels C and D, each column represents a sample. Columns are labeled using the convention [Cell Line]_[Condition][Replicate], where cell lines are designated as F1, F2, and F3 for three independent IPF patient-derived fibroblast lines, and C1, C2, and C3 for three independent control fibroblast lines. H1–H3 denote three technical replicates cultured under hypoxia (1% O_2_, 48 h), and N1–N3 denote three technical replicates cultured under normoxia (21% O_2_, 48 h). Color-coded annotation bars above each heatmap indicate the cell line identity and the experimental condition (orange = hypoxia; purple = normoxia). Rows represent genes and are clustered using hierarchical clustering (Euclidean distance, Ward’s D2 method). Color intensity reflects row-normalized z-scores of log_2_ expression values (blue = down-regulated; red = up-regulated; white = no change). Samples are grouped by cell line to allow assessment of both intra-cell line reproducibility (consistency among H1–H3 or N1–N3 replicates within a cell line) and inter-cell line concordance (agreement across F1–F3 or C1–C3). An alternative arrangement with samples ordered by replicate number is provided in Supplementary Figure S4. DEGs were identified using adj. *p*-value < 0.05, |log_2_FC| ≥ 1, protein-coding transcripts only (NM_ accessions). Heatmaps with gene symbols are shown in Supplementary Figure S5. (**E**) Gene Ontology (GO) biological process enrichment analysis of the differentially expressed genes in control fibroblasts under hypoxia. (**F**) GO biological process enrichment analysis of the differentially expressed genes in IPF fibroblasts under hypoxia. In panels (**E**,**F**), each bar corresponds to a significantly enriched GO biological process term, ordered by the statistical significance of the enrichment.
