# Supplementary material for: Effect of Hypoxia in the Transcriptomic Profile of Lung Fibroblasts from Idiopathic Pulmonary Fibrosis
Source: Cells. 2022 Sep 27;11(19):3014. doi: 10.3390/cells11193014 (PMC9564151; doi:10.3390/cells11193014)
Supplement: Supplementary file 1 [file cells-11-03014-s001.zip › Supplementary_Figure S1.pdf]

			Contrasts											
			Control						IPF					
			C1	C2	C3	C1	C2	C3	F1	F2	F3	F1	F2	F3
			Normoxia			Hipoxia			Normoxia			Hipoxia		
Control	C1	Normoxia												
	C2													
	C3													
	C1	Hipoxia	Control Hipoxia vs Control Normoxia											
	C2													
	C3													
IPF	F1	Normoxia												
	F2													
	F3													
	F1	Hipoxia							IPF Hipoxia vs IPF Normoxia					
	F2													
	F3													

			Contrasts											
			Control						IPF					
			C1	C2	C3	C1	C2	C3	F1	F2	F3	F1	F2	F3
			Normoxia			Hipoxia			Normoxia			Hipoxia		
Control	C1	Normoxia												
	C2													
	C3													
	C1	Hipoxia	C1 H vs C1 N											
	C2			C2 H vs C2 N										
	C3				C3 H vs C3 N									
IPF	F1	Normoxia												
	F2													
	F3													
	F1	Hipoxia							F1 H vs F1 N					
	F2									F2 H vs F2 N				
	F3										F3 H vs F3 N			
