# Supplementary figures and images for: Effect of Hypoxia in the Transcriptomic Profile of Lung Fibroblasts from Idiopathic Pulmonary Fibrosis
Source: Cells. 2022 Sep 27;11(19):3014. doi: 10.3390/cells11193014 (PMC9564151; doi:10.3390/cells11193014)

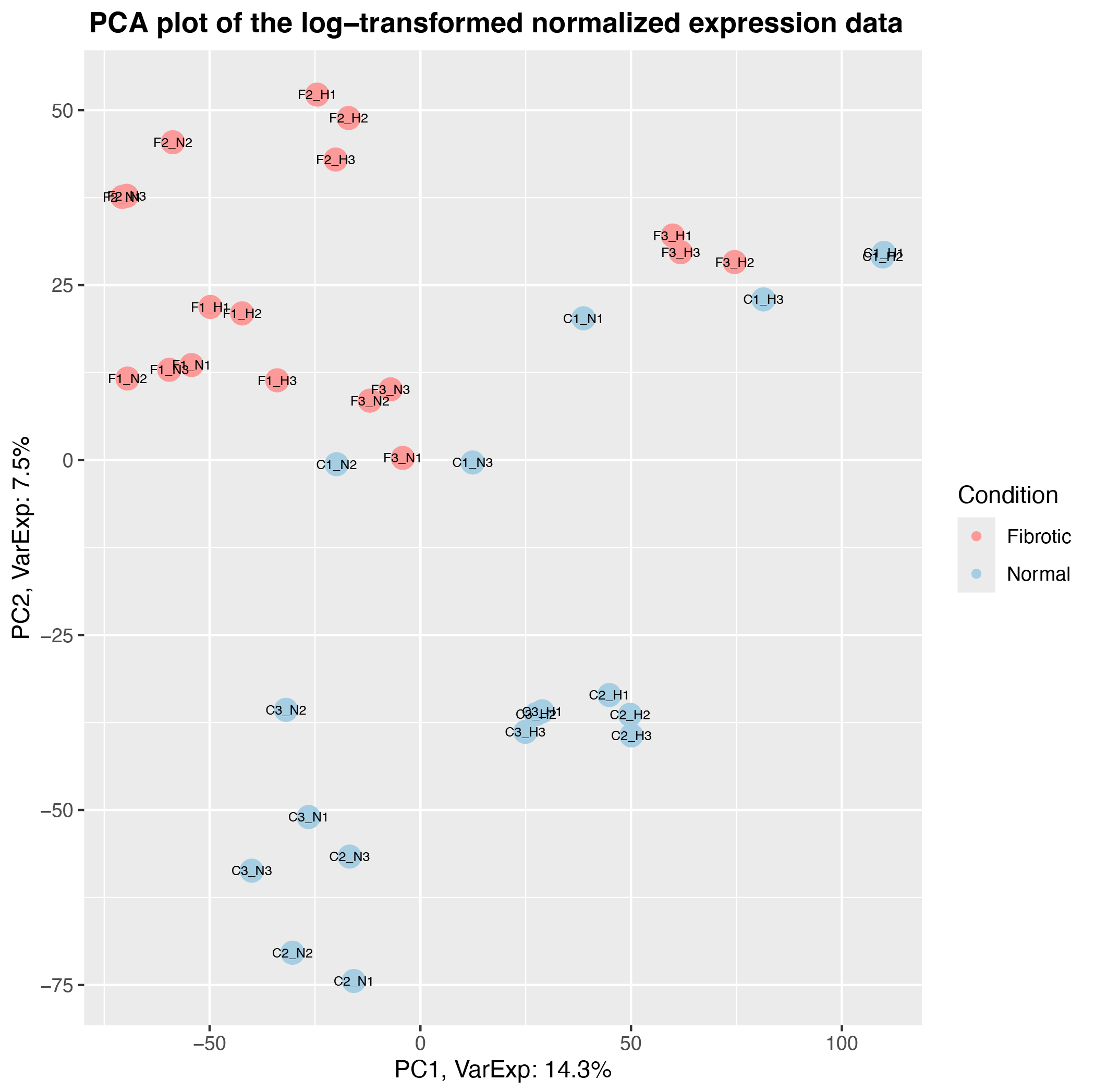

Supplement: Supplementary file 1 [file cells-11-03014-s001.zip › Supplementary_Figure S2.png]

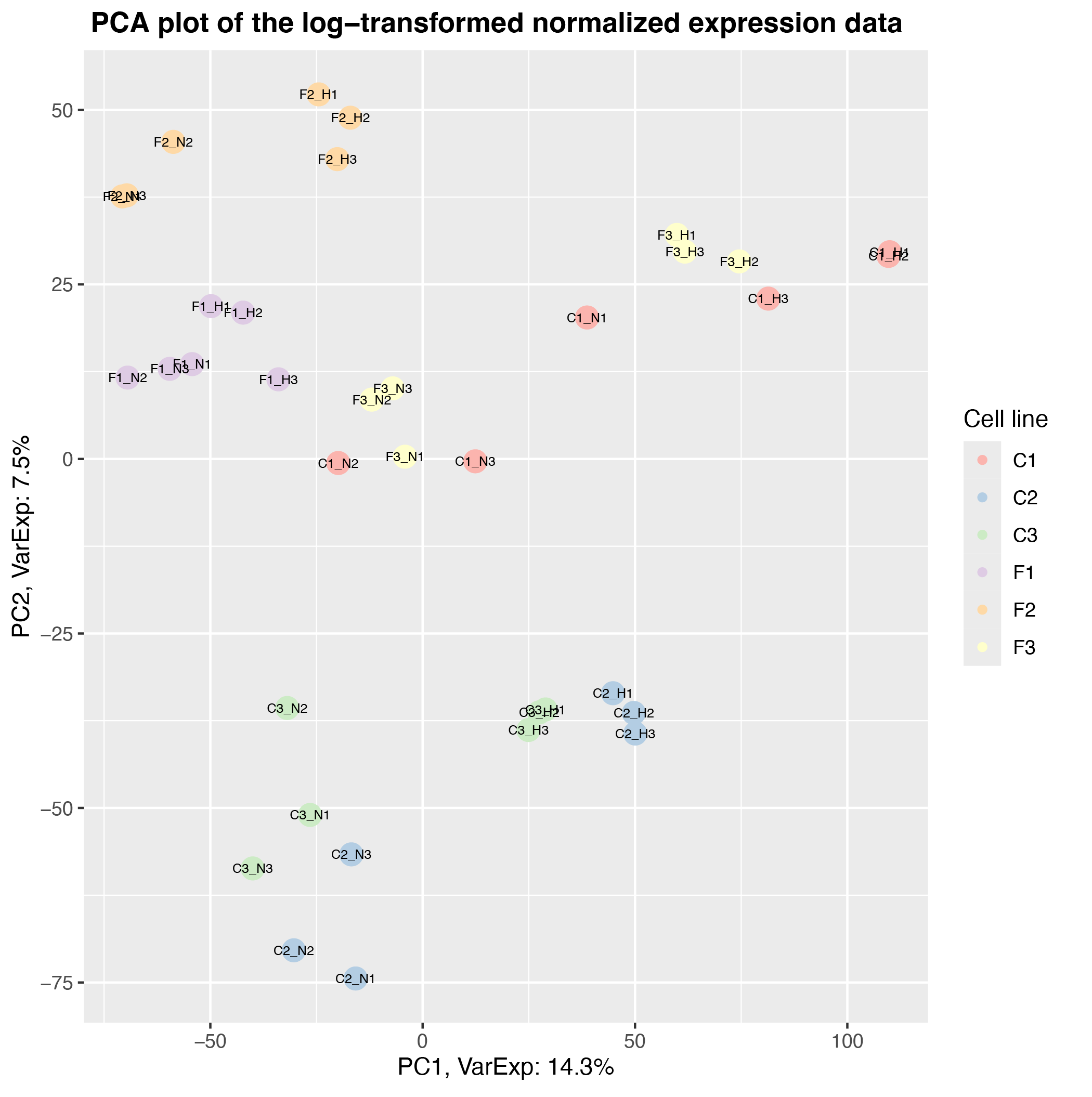

Supplement: Supplementary file 1 [file cells-11-03014-s001.zip › Supplementary_Figure S3.png]

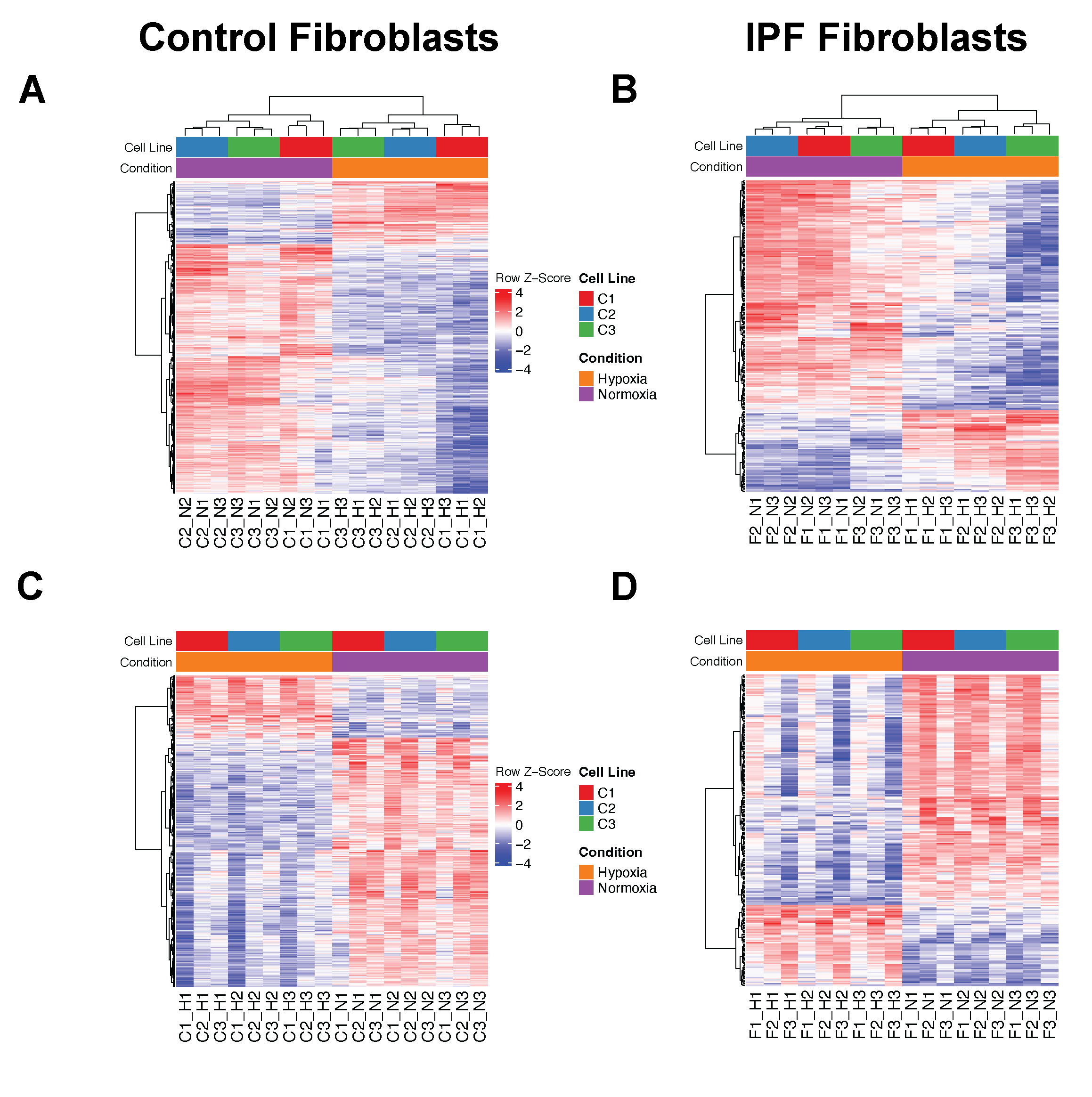

Supplement: Supplementary file 1 [file cells-11-03014-s001.zip › Supplementary_Figure S4.png]
