# Supplementary material for: Effect of Hypoxia in the Transcriptomic Profile of Lung Fibroblasts from Idiopathic Pulmonary Fibrosis
Source: Cells. 2022 Sep 27;11(19):3014. doi: 10.3390/cells11193014 (PMC9564151; doi:10.3390/cells11193014)
Supplement: Supplementary file 1 [file Supplementary_Figure S5.pdf › Supplementary_Figure S5.pdf]

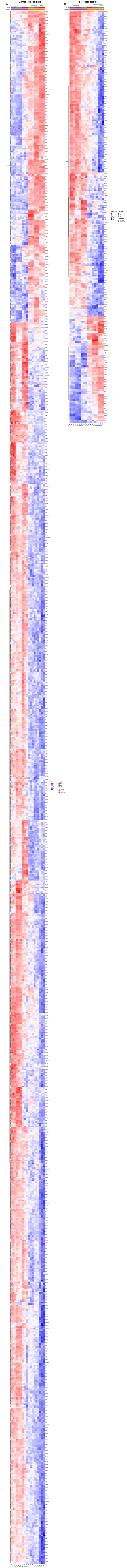
A
Control Fibroblasts
B
IPF Fibroblasts
